# The Effect of Size and Taper of Apical Preparation in Reducing Intra-Canal Bacteria: A Quantitative SEM Study

**Published:** 2013-12-24

**Authors:** Nahid Mohammadzadeh Akhlaghi, Nahid Rahimifard, Amirabbas Moshari, Mehdi Vatanpour, Soheila Darmiani

**Affiliations:** a *Department of Endodontics, Dental Branch, Islamic Azad University, Tehran, Iran; *; b *Department of Microbiology; Food and Drug Control Laboratories; Food And Drug Laboratories Research Center; Ministry of Health and Medical Education, Tehran, Iran;*; c *Department of Endodontics, Dental School, Shahid Beheshti University of Medical Sciences, Tehran, Iran*

**Keywords:** Apical Size, Bacteria, Root Canal Therapy, Scanning Electron Microscopy

## Abstract

**Introduction: **Bacteria and their byproducts are major etiologic factors in endodontic diseases. Prevention or reduction of root canal bacterial contamination is the main aim of endodontic treatment. The purpose of this *in vitro* study was to evaluate the effect of size and taper of master apical file (MAF) in reducing bacteria from the apical third of the curved canals using a quantitative scanning electron microscope (SEM) study.** Methods and Materials:** Eighty-nine human mandibular first molars with curved MB canals (20^º^-35^º^) were divided into one control group (*n*=5) (without rotary instrumentation) and 6 experimental groups (*n*=14). The canals were prepared using RaCe rotary files to the MAF sizes 25/0.04, 25/0.06, 30/0.04, 30/0.06, 35/0.04 and 35/0.06, in groups 1 to 6, respectively. All the experimental groups were finally rinsed with 2 mL of 17% EDTA followed by 3 mL of 5.25% NaOCl. The mesial roots were split longitudinally. Remaining bacteria in the apical third of MB canals were evaluated using SEM (2000×). Data analysis was performed using one way ANOVA with Tukey’s post hoc test. The level of significance was set at 0.05. **Results:** All the experimental groups showed significant bacterial reduction (*P*<0.001). Although the greater size and/or taper resulted in decrease in bacteria, differences between the groups were not significant. **Conclusion:** Based on this *in vitro *study the MAF #25/0.04 had no significant difference compared to other groups with greater apical size/taper; all groups could effectively reduce intra-canal bacteria.

## Introduction

Bacteria and their byproducts are the major etiologic factors in endodontic diseases [1-3]. Therefore prevention or reduction of root canal bacterial contamination is the main purpose of endodontic treatment [4, 5]. This goal will be best reached with appropriate chemo-mechanical preparation [6, 7], along with conserving as much tooth structure as possible and maintaining the original canal geometry [8]. Therefore, it is desirable to reduce as much bacterial contamination as possible with minimum mechanical preparation and proper chemical disinfection.

Preparation with larger instruments results in more volume of irrigation reaching the apical region. On the other hand larger instruments are less flexible and do not stay centered in the canal. It’s more of a problem in curved canals, which results in unnecessary dentin removal on one side of the canal, leaving untouched dentin on the other side of the canal walls [9, 10].

It has been shown that irrigation will be more effective when canal preparation size and taper is larger [5, 11, 12]. Different amounts of apical enlargement have been suggested by different studies [11-13]. Some studies found that apical preparation up to size 30 could effectively clean root canals [13, 14]. Preparation to sizes larger than 30 or 35 was found to be required for NaOCl to effectively eliminate bacteria in another study [15]. One study suggested apical enlargement to 40/0.04 as a good balance between preservation of tooth structure and maximum volume of irrigation using a negative pressure irrigation system in apical third [16]. Although Elayouti *et al.* emphasized on keeping the apical enlargement to minimum size required for effective irrigation [9], Akhlaghi *et al.* concluded that apical preparation even to file #30/0.10 left the minimum required root wall thickness of 0.3 mm [10].

Several studies have used Scanning Electron Microscopy (SEM) to evaluate biofilm quality [17, 18], and detection of bacterial presence on canal walls [14, 19-21], or bacterial penetration into dentinal tubules [22-24]. Also SEM micrographs have been used to assess the thickness of smear layer on canal walls [13]. SEM micrographs can be used to compare bacterial counting in randomly selected sections of the root canal wall. This method can enumerate attached bacteria on root canal surfaces.

To date, no microbiological study has evaluated the simultaneous effect of size and taper of master apical file (MAF) on bacterial reduction from the canal. The purpose of this *in vitro* study was to compare the effect of final preparation size and taper in reducing bacteria from curved root canals using a quantitative SEM method.

## Material and Methods

***Sample collection***

In this experimental study the fully developed mandibular first molars extracted for periodontal reasons were collected and disinfected by immersion in 5.25% NaOCl (Golrang, Tehran, Iran) for 1 h. After providing the periapical radiographs, teeth with external or internal root resorption, visible cracks, fractures, caries, calcification and previous root canal treatment were excluded. After preparing the access cavity, the presence of the two separate mesial canals (*i.e. *Type III) and the patency of the mesiobuccal canals were confirmed, and the teeth with an apical constriction diameter wider than a #15 file in mesial roots were excluded. The degree of curvature of mesiobuccal canal was determined according to Schneider technique in both buccolingual and mesiodistal directions [25]. Only canals with curvatures of 20^º^-35^º^ were included. Working length (WL) was determined by inserting a #10 K-file (Dentsply Maillefer, Ballaigues, Switzerland) until the tip emerged from the apical foramen and then it was subtracted 1 mm from this length. A total number of 89 teeth were decoronated to obtain a standardized root length of 18 mm using #10 K-file, with a WL of 17 mm.

***Sterilization***

Teeth were autoclaved at 121^° ^C with +25 psi pressure for 30 min in separate vials. Six teeth were randomly selected to confirm sterilization. Three teeth were immersed in three separate vials containing BHI (brain-heart infusion) broth (Difco Laboratories, Detroit, MI) and three in separate vials of thioglycollate culture medium (TGC, Difco Laboratories, Detroit, MI, USA). Each vial was incubated for 14 days at 37^°^ C. A clear culture medium after incubation confirmed sterility.

***Bacterial inoculation***

Each vial was opened in a laminar air flow cabinet –under sterile conditions– and a fresh suspension of *Enterococcus. faecalis* *(E. faecalis*, ATCC 29212) with a turbidity equivalent to a 0.5 McFarland turbidity standard was introduced into each mesiobuccal canal using #15 K-File. Each tooth was immersed in a pre-sterilized vial containing sterile BHI broth. Vials were incubated for 48 h at 37^°^ C. Presence of turbidity confirmed bacterial contamination in all samples.

***Experimental and control groups***

By the table of random numbers, the teeth were randomly divided into 6 experimental groups (*n*=14) and a control group (*n*=5), the later without rotary instrumentation.

Rotary preparation was performed under sterile conditions in a laminar air flow cabinet, using sterilized RaCe instruments (FKG Dentaire, La-Chaux-de Fonds, Switzerland) and a motor controller device (X-SMART, Dentsply Maillefer, Ballaigues, Switzerland) according to the manufacturer’s instructions. For all samples coronal pre-flaring was done using sizes 40/0.10‚ 35/0.08 and 30/0.06 subsequently. Canal preparation to the WL was done as follows:

*Group 1*: 25/0.04 

*Group 2*: 25/0.04 ‚ 25/0.06

*Group 3*: 25/0.04‚ 30/0.04

*Group 4*: 25/0.04‚ 30/0.04‚ 30/0.06 

*Group 5*: 25/0.04 ‚30/0.04‚ 35/0.04

*Group 6*: 25/0.04‚ 30/0.04‚ 35/0.04‚ 35/0.06

A third-year post-graduate student of endodontics prepared all the canals. Each rotary instrument was used for preparation of five canals. Each instrument was applied for 5 sec to the WL with an anti-curvature filing method. Subsequent to each rotary file, the canal was irrigated with 2 mL of 1% NaOCl using a 28 gauge needle (Dentsply Rinn, Elgin, IL) and canal patency was checked using #10 K-File. At the end of coronal pre-flaring process, the irrigation needle was passively inserted from the coronal to middle third. Also during apical preparation sequence, the needle was placed to the apical 3 mm. In all the experimental groups, final irrigation was performed using 2 mL of 17% EDTA solution (Roth International Ltd., Chicago, IL) and 3 mL of 5.25% NaOCl. Each solution was left in the canal for one min followed by final flushing using 5 mL sterilized distilled water to eliminate the irrigation solutions from the root canal. In the control group only 5 mL of normal saline was used.

***Preparation for SEM evaluation***

The samples were prepared for SEM evaluation in the following manner. A groove on each of the buccal and lingual aspects of the mesial root was prepared without entering the canal space. In addition, two grooves were prepared in the apical 5 mm of the mesial and distal walls and the root was separated and then split longitudinally.

**Figure 1:**
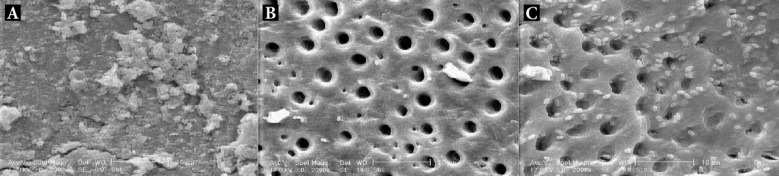
*A)* SEM photographs of a control group and, some of the experimental groups; *B)* MAF #35/0.06; *C)* MAF #25/0.06

Half of each sample was randomly chosen, placed in 2% glutaraldehyde for 24 h and then rinsed 3 times with a sodium cacodylate buffered solution (0.1 M, pH=7.2). After incubation in osmium tetroxide for 1 h, the samples were desiccated with ascending concentrations of ethyl alcohol (30-100%), placed in a desiccator for 24 h and mounted on a metallic stub. After coating the samples with 20 μ of gold, SEM photomicrographs were taken using a back scatter mode (2000×; XL30, Philips, Holland). A third-year post-graduate student of endodontics observed the SEM photographs. Each image was divided into nine equal squares, and the bacteria in the upper right quadrant were counted (Figure1).

**Table 1:** The mean number of bacteria in each SEM micrograph after canal preparation in control and experimental groups

**Size/taper of MAF **	**Quantity of bacteria (SD)**
**25/0.04**	177.53 (51.36)
**25/0.06**	167 (70.32)
**30/0.04**	166 (62.36)
**30/0.06**	165.69 (72.94)
**35/0.04**	165.61 (67.73)
**35/0.06**	163.76 (75.47)
**Control group **	700

***Statistical analysis***

Data analysis was performed using the Kruskal–Wallis test. The level of significance was set at 0.05.

## Results

All the experimental groups showed significant bacterial reduction compared with the control group (Table 1).

Although the greater size and/or taper led to more decrease in amount of bacteria, the differences between the groups with similar size and different tapers, and between the groups with the similar taper and different sizes were not significant (*P*>0.05).

No file deformity, broken instruments and apical perforation occurred during instrumentation. Some of the SEM micrographs showed non-coccoid bacterial contamination that seems to have happened as a result of sample preparation and handling for SEM evaluation. All of these contaminations were spirochete-like bacteria and were not included in the counting results.

## Discussion

The present *in vitro *study compared the effect of different sizes and tapers of MAF on decreasing the bacterial count in curved mesiobuccal canals of mandibular first molars, using RaCe rotary files. There was no significant difference among the experimental groups with the same sizes and different tapers and neither between the groups having identical tapers and different sizes (*P*>0.05), but there was a significant difference between each experimental group compared to the control group (*P*<0.001).

This study like some other studies were performed on curved mesiobuccal canals of mandibular first molars [9, 10, 13-16]. Shuping *et al.* [15]and Siqueira *et al.* [4] conducted their studies on premolar teeth with at least one straight canal, thus did not assess the challenge of bacterial reduction in curved and narrow canals. The former was an *in vivo* study. Some other similar studies did not mention the degree of curvature [11, 16, 26]. Since canal curvature can have an effect on depth of needle and irrigant penetration and also canal preparation, the results of above mentioned studies could not be generalized to most clinical situations [27, 28]. Because of the important effect of curvature on the canal preparation, we considered using curved canals (20^°^ to 35^°^) like some other studies [9, 10]. According to the several previous studies, the debridement ability of RaCe is superior to some other NiTi rotary systems [29-31]. So RaCe rotary instruments were used in this study.

Irrigant type and method of irrigation in our study was similar to some other studies [11-13, 32]. But compared to Khademi *et al.*, we used a greater volume of irrigating solution [13]. In some studies, using lower concentrations of NaOCl led to acceptable results [5, 15]. According to the results by Giardino *et al.* [33] and Soares *et al.* [34], the best concentration of NaOCl for eliminating the bacterial biofilm from root canal is 5.25%. In addition, the best regimen for acceptable elimination of smear layer is using 17% EDTA for 1 min followed by a 3-5 mL rinse with 5.25% NaOCl [35, 36]. Thus we used this method as final irrigation before rinsing with pre-sterilized distilled water.

We used SEM micrographs instead of culture based bacterial counting which has been used in some other studies [1, 2, 14, 15]. This method has the advantage of being more accurate and includes attached bacteria to the canal walls instead of non-attached ones. In culture based methods, the bacteria detached from the canal walls are absorbed by the paper point and this does not provide information about what remains on the root canal walls. It is not clear whether the microorganisms that are absorbed into the paper point are always representative of those involved in the infectious process, or not [37]. Most clinical problems are due to attached but not floating bacteria. So this method can be more reliable in predicting the remained bacteria on the canal walls.

In this study, the difference between the experimental and the control groups was significant. Furthermore, increasing the size and/or taper of MAF did not result in significant decrease of remaining bacteria among the experimental groups. The control group had significantly more bacteria than all other experimental groups.

Card *et al*. used 2 mL of 1% NaOCl as irrigant and showed that increasing apical preparation size led to more bacterial reduction [5]. This was more obvious in teeth with a single straight canal. Shuping *et al.* used 1.25% NaOCl without reporting the volume [15]. They confirmed the results of Card *et al.* and stated that bacterial reduction would be much more effective when NaOCl is used as irrigating solution.

Results of Siqueira *et al.* showed that increasing the size of apical file to #40 could reduce the bacterial count significantly more than smaller sizes [4]. They used 7 mL of 0.85% normal saline as irrigating solution to eliminate the chemical aspect of chemo-mechanical preparation, and thus assessed the effect of mechanical instrumentation per se. In the present study increasing the size of MAF resulted in less remaining bacteria, but because of using NaOCl, this bacterial reduction was not statistically significant among the experimental groups.

Contrary to the results of the present study, Wu and Wesselink concluded that preparation of canals in molars with #40 hand files leaves significantly less bacteria than smaller files [38]. Dalton *et al.* concluded that preparing the canal with larger sizes lead to more disinfection, but even larger sizes could not render the root canal bacteria free [1]. In this study, MAF size of 25/0.04 did not show a significant difference compared to the other experimental groups. Elayouti *et al.* suggested to keep the apical size of curved canals as minimal as possible, provided that a sufficient irrigation is feasible [9].

## Conclusion

Based on the results of this* in vitro* study, although the size of 25/0.04 had no significant difference on bacterial reduction compared to greater sizes and/or tapers, further studies are required to elaborate the effect of final apical size/taper on the penetration and volume of irrigants within the canals, as well as the amount of remaining smear layer/debris on the canal surfaces and the amount of remaining dentin thickness, to determine the optimum size/taper of MAF.
